# Asymmetric Diffraction in Plasmonic Meta-Gratings Using an IT-Shaped Nanoslit Array

**DOI:** 10.3390/s21124097

**Published:** 2021-06-14

**Authors:** Hee-Dong Jeong, Seong-Won Moon, Seung-Yeol Lee

**Affiliations:** 1 School of Electronic and Electrical Engineering, College of IT Engineering, Kyungpook National University, Daegu 41566, Korea; heedong@knu.ac.kr (H.-D.J.); swmoon@postech.ac.kr (S.-W.M.); 2 Department of Mechanical Engineering, Pohang University of Science and Technology, Pohang 37673, Korea

**Keywords:** asymmetric diffraction, metasurface, nanostructured optical filter, non-reciprocal

## Abstract

Diffraction is a fundamental phenomenon that reveals the wave nature of light. When a plane wave is transmitted or reflected from a grating or other periodic structures, diffracted light waves propagate at several angles that are specified by the period of the given structure. When the optical period is shorter than the wavelength, constructive interference of diffracted light rays from the subwavelength-scale grating forms a uniform plane wave. Many studies have shown that through the appropriate design of meta-atom geometry, metasurfaces can be used to control light properties. However, most semitransparent metasurfaces are designed to perform symmetric operation with regard to diffraction, meaning that light diffraction occurs identically for front- and back-side illumination. We propose a simple single-layer plasmonic metasurface that achieves asymmetric diffraction by optimizing the transmission phase from two types of nanoslits with I- and T-shaped structures. As the proposed structure is designed to have a different effective period for each observation side, it is either diffractive or nondiffractive depending on the direction of observation. The designed structure exhibits a diffraction angle of 54°, which can be further tuned by applying different period conditions. We expect the proposed asymmetric diffraction meta-grating to have great potential for the miniaturized optical diffraction control systems in the infrared band and compact optical diffraction filters for integrated optics.

## 1. Introduction

Because of recent growth of industrial fields related to digital holographic and compact integrated optical device technologies, the use of light diffraction as a key technology has become tremendously important. This includes light display via spatial light modulators, image sensing and the precise extraction of external information through sophisticated lens systems [1,2,3,4,5,6,7,8,9,10]. Diffraction is a representative characteristic of light waves, and it is well known that when a plane wave is transmitted or reflected in a gridded periodic structure, multiple degrees of diffraction occur at specific angles based on the diffraction law. Conversely, when the period of a system is below the diffraction limit, constructive interference of diffracted light rays from the subwavelength-scale grating forms a uniform plane wave. The light transmission and reflection characteristics are effectively determined by the geometry of the subwavelength unit structure, which is a key concept for building metasurfaces with sophisticated functions [11,12,13,14,15].

A metasurface is an artificially designed surface with a unit-cell geometry made up of meta-atoms, which are designed by periodically arranging the structure at the subwavelength scale to precisely control its general light-interaction properties, such as reflection/refraction [16,17], polarization [18,19,20], and nonlinear response [21,22]. Based on the advent of the metasurface, various issues that were difficult to overcome with geometric optics alone have been resolved, such as the development of super-resolution lenses beyond the diffraction limit and aberration-free metalenses for imaging systems [23,24,25,26]. Metasurfaces are generally fabricated via a well-known semiconductor fabrication process, and they can be made to be very compact, thereby assisting in the miniaturization of optical systems. Increasing numbers of studies are being reported on the application of metasurfaces to practical technologies instead of conventional optics, such as to see-through metalenses for AR and wide-viewing-angle digital holograms [27,28,29,30,31].

Although various types of metasurfaces for customizing the optical properties of light have been reported, most of them have symmetrical transmission, producing the same output on the front and back sides of the structure. Breaking such a symmetry to achieve what is often called the asymmetrical transmission of light may be an important technique for integrated optics, optical telecommunications, and AR displays owing to its role in optical isolation [32,33,34].

However, it is well known that an ideal level of optical isolation can only be achieved using a medium with nonreciprocal, nonlinear or chiral metamaterials, which require bulky optics or a complex fabrication process, respectively [35,36]. Moreover, recently, various studies have reported breaking the traditional rules of optical symmetry using metasurfaces, although they have not used nonreciprocal materials to completely violate the Lorentz reciprocity theorem [37].

For example, studies have reported asymmetric optical properties for different directions by optimizing the geometric conditions of metasurfaces, such as an asymmetric-diffraction optical device using a difference in phase delay caused by tuning the parameter of the depth of the groove [38] or an asymmetric propagation device using the difference in intensity and phase delay of electromagnetic waves caused by the lossy planar chiral structure [39]. Additionally, asymmetric transmission studies using a nanopolymerization technique, such as multichannel polarization conversion elements using anisotropic diffraction stripe structures in which nematic molecule materials are homogeneously arranged, have been reported [40].

Moreover, a study by Lawrence et al. demonstrated the non-reciprocal manipulation of near-infrared (NIR) light with ultrathin metasurfaces [41]. The proposed structure exhibited one-way transmission through a thin periodic silicon metasurface using high-quality-factor resonance dependent on the Kerr effect [42,43]. However, such an approach generally relies on ultrafast optical nonlinearities to achieve fast modulation rates. Therefore, high power is required, leading to a disadvantage in terms of power consumption.

Further, Frese et al. demonstrated a two-layer metasurface system that breaks the spatial symmetry of propagation, resulting in distinct bidirectional holographic image generation [44]. Their proposed structure has adjustable asymmetric transmission properties with full phase and amplitude modulation. This method leads to a high degree of freedom regarding tuning, but because the system is composed of two layers, it is bulky and expensive and aligning the two layers requires a high-end fabrication technique.

As previously outlined, metasurfaces with asymmetric transmission properties are being studied using various approaches. However, to the best of our knowledge, a device with a single-layer thin-film structure capable of asymmetric diffraction has not yet been reported.

In this study, we propose a novel design for a plasmonic metasurface as part of an asymmetric diffraction filter, for which the presence or absence of diffracted light can be selected according to the direction of illumination. Because the proposed structure is not designed as a nonreciprocal material or structure, it cannot completely block the output in the opposite direction. However, the proposed method enables asymmetric diffraction characteristics to be obtained without breaking the Lorentz reciprocity theorem.

In the proposed structure, the unit cell of meta-atoms is composed of closely organized I- and T-shaped slits [45,46,47,48], with the “IT-shaped” unit cell being periodically arranged. The key principle of the proposed structure is to create a difference in effective period. In particular, it is designed to be shorter than the incident wavelength for the forward illumination case, defined as that in which light illumination is through the wide part of T-shaped slits. Consequently, the plane wave is transmitted without diffraction. By contrast, the effective period for the backward illumination case is designed to be longer than the incident wavelength, meaning the incident light is diffracted according to the diffraction law. Because the proposed structure is able to exhibit these two optical properties with only a single layer, the optical system can be compactly designed and fabricated at a low cost. Further, the proposed diffraction filter has a diffraction angle of approximately 54° for the NIR wavelength, though the diffraction angle can be selectively adjusted by changing the period between meta-atoms. In the next section, we first explain the configuration of the proposed structure. Then, we present the results regarding the simulated optical properties. Details of the optimization of the proposed device follow, after which the conclusion of our work are summarized.

## 2. Basic Principle of the Proposed Structure

**Figure 1:**
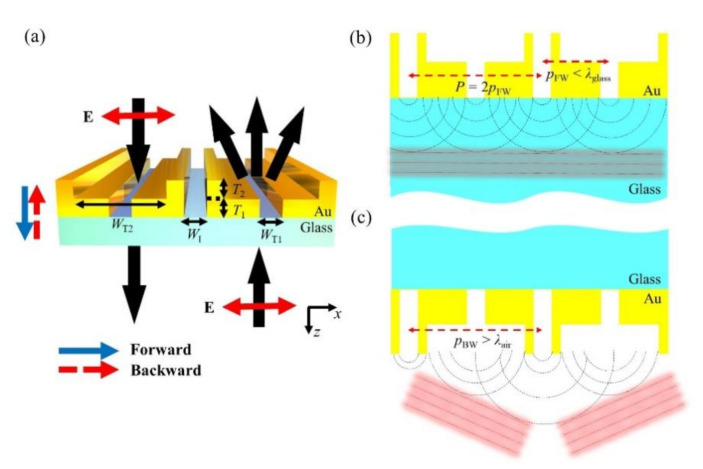
(**a**) Schematic of the proposed structure. The red dashed and blue solid lines indicate the light illumination directions with and without diffraction, respectively. (**b**,**c**) Schematic explaining the asymmetric diffraction of the proposed structure in the (**b**) forward and (**c**) backward directions.

A schematic of the proposed asymmetric optical diffraction structure is shown in Figure 1a. The unit cell of the proposed meta-grating structure comprises two adjacent nanoslits, one being a normal slit (I-shaped slit) and the other being a trenched slit (T-shaped slit). These are patterned on a gold film, which is deposited on a SiO_2_ substrate. Here, the width of the I-shaped slit is denoted by *W*_I_, and the narrower and wider areas of the T-shaped slit are denoted by *W*_T1_ and *W*_T2_, respectively. Similarly, the thicknesses of the narrower and wider areas of the T-shaped slit are denoted as *T*_1_ and *T*_2_, respectively. *P* is the physical period of the unit cell, which indicates the intervals between I-shaped slits, with the pattern being periodically arranged.

The key concept behind obtaining asymmetric optical diffraction according to the incident light direction is to design different optical periods for forward and backward illumination (*p*_FW_ and *p*_BW_, respectively) at the output port of the proposed structure. For the forward illumination case, *p*_FW_ is designed to be less than *λ*_glass_ (Figure 1b) by optimizing the complex amplitudes of the out-coupled light from the I- and T-shaped slits to have identical values, so that the optical period of forward illumination is half of the physical period (*p*_FW_ = *P*/2). Because the light transmitted through the output port of the slit generates a new point light source with an effective period shorter than the wavelength, the wavefront generated by these sources is nondiffractive. At the same time, as shown in Figure 1c, *p*_BW_ is designed to be longer than *λ*_air_ because the complex amplitudes of out-coupled light are generally different from each other. Therefore, when light illumination is from the backward direction, the wavefronts generated by the I- and T-shaped slits are different from each other (*p*_BW_ = *P*), resulting in strong diffraction. Such an asymmetric diffraction depending on the illumination direction can be achieved by setting *P* as $\lambda_{\mathrm{air}}<P<2\lambda_{\mathrm{glass}}$, and the first-order diffraction angle for backward illumination can be expressed as:

(1) $$\sin\theta_{diff}=\pm \frac{\lambda_{\mathrm{air}}}{P}$$

## 3. Results and Discussion

**Figure 2:**
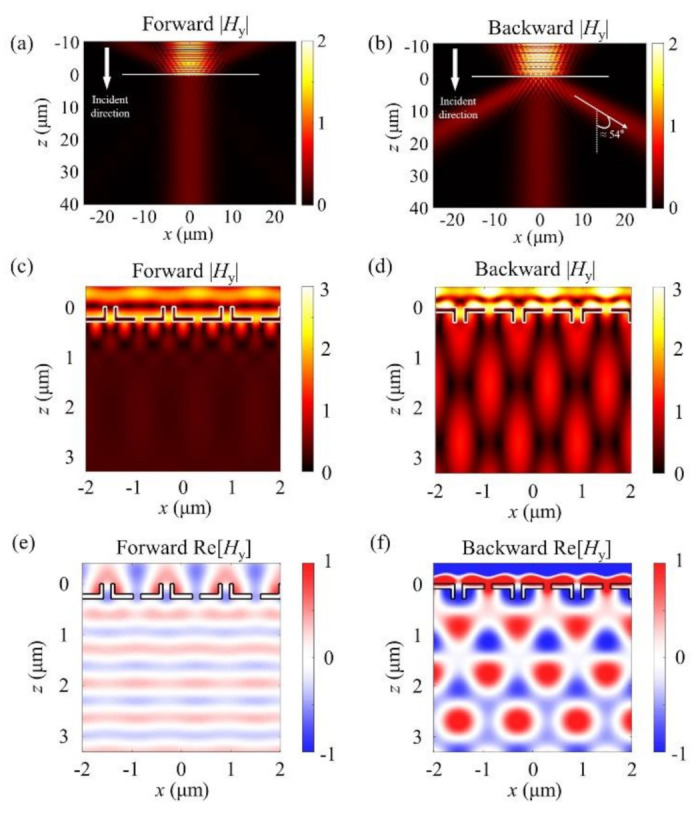
(**a**,**b**) *H*_y_ field distribution of (**a**) forward and (**b**) backward illumination cases when the Gaussian beam is normally incident on the proposed structure. The white arrow indicates the direction of the incident light, and the horizontal line indicates the location of the proposed structure. (**c**,**d**) Absolute value of the magnetic field in the (**c**) forward and (**d**) backward illumination cases near the nanoslit array. (**e**,**f**) Calculated real part of the magnetic field distribution (**e**) without diffraction (Forward Re[*H*_y_]) and (**f**) with diffraction (Backward Re[*H*_y_]).

For our numerical analysis, we used the rigorous coupled-wave analysis (RCWA) method to calculate the full electromagnetic field distribution and diffraction efficiencies [49]. The boundaries were selected as periodic in the *x*-direction and open in the *z*-direction. The structure was assumed to be periodic and infinite in the *x*- and *y*-directions, respectively. We used NIR light with a wavelength of 980 nm, and the light was polarized perpendicular to the slit array. For the sake of clarity, we first show the results of the proposed structure under optimized conditions. Figure 2a,b show the simulated *H*_y_ field distribution when a Gaussian beam illuminates the structure in the forward and backward directions, respectively.

The reason we focused on the *H*_y_ field is that the simulation conditions included no *y*-direction dependency. Therefore, only the *E*_x_, *H*_y_, and *E*_z_ components exist, and the two electric field components (*E*_x_ and *E*_z_) could be directly calculated from the *H*_y_ field. In these simulations, the parameters *W*_I_, *W*_T1_, *W*_T2_, *T*_1_, and *T*_2_ were set to 150, 150, 900, 100, and 200 nm, respectively. With these parametric conditions, the complex amplitudes of the I- and T-shaped nanoslits were identical for all forward directions, while those for backward directions were designed to have a phase difference of π to maximize the diffraction efficiency. The complex amplitude can be expressed as, for example, *H*_y_ = |*H*_y_|exp(∠*H*_y_), where |*H*_y_| is the absolute amplitude of the *H*_y_ field, and ∠*H*_y_ is the phase of that field.

As shown in Figure 2a, in the forward direction, light passes through the entire surface of the proposed structure without diffraction. By contrast, Figure 2b clearly shows that light transmitted through the back side is significantly diffracted, at an angle of approximately 54°, agreeing well with the calculation results from Equation (1).

To further investigate the field distribution of the meta-grating structure, we observed the near-field characteristics, as presented in Figure 2c–f. Figure 2c,d show the absolute amplitude of the *H*_y_ field for each illumination direction, while Figure 2e,f show the real part of the *H*_y_ field near the proposed nanoslit array. In Figure 2c,e, because the light passing through the proposed structure in the forward direction undergoes no diffraction, the absolute amplitude of the transmitted light is kept constant and the real part proceeds in the form of plane waves. It is worth noting that the out-coupled phases of the I- and T-shaped slits are almost identical, clearly indicating that the effective optical period is not the same as the physical period, instead being half of the physical period. By contrast, in the backward direction, because there is a large difference in the phase of the light passing through the two types of slits on the output side, the absolute amplitude of the transmitted light is not constant, as shown in Figure 2d. Such a phase difference is more clearly exhibited in Figure 2f, which shows the positive phase for the T-shaped slits in red and the phase for the I-shaped slits in blue. 

**Figure 3:**
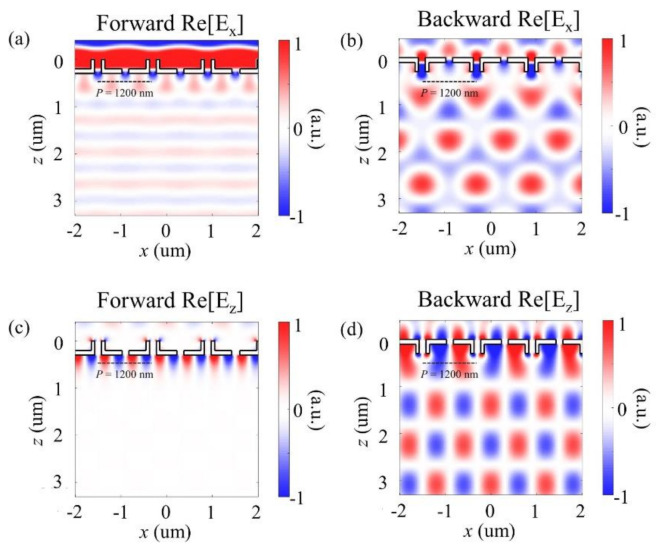
Real part of the *E*_x_ field distribution for (**a**) forward (Forward Re[*E*_x_]) and (**b**) backward illumination cases (Backward Re[*E*_x_]). The simulated real part of the *E*_z_ propagate (**c**) without diffraction (Forward Re[*E*_z_]) and (**d**) with diffraction (Backward Re[*E*_z_]) illumination on the proposed structure.

In addition, Figure 3 shows the *E*_x_ and *E*_z_ field distribution for the forward and backward illumination cases. As shown in Figure 3a, similar to the case of the magnetic field, the constructive interference of diffracted light rays from the IT-slit array formed a uniform plane wave because it was designed to have no phase difference between the I- and T-shaped slits. Meanwhile, as shown in Figure 3b, in the backward direction, an interference pattern could be obtained from the phase difference between the I- and T-shaped slits. Moreover, Figure 3c depicts an image of the calculated real part of the *E*_z_ component in the forward direction. Because there was no *E*_z_ component in a plane wave propagating at an angle of 0°, only the near field was observed. In the backward direction (Figure 3d), the interference pattern generated by the wavefronts propagated with the 1st ordered diffraction angles.

Now, we will discuss the process for determining the optimal conditions in more detail. Because a large number of parameters can be controlled, we first needed to appropriately classify the parameters to be controlled or fixed to efficiently achieve our goal. First, we assumed that it would be best to match the values of *W*_I_ and *W*_T1_, as the amount of out-coupled light from the I- and T-shaped slits is generally proportional to these values, and it is necessary to match both the amplitude and phase of these slits in the forward illumination case. Second, *W*_I_ and *W*_T1_ had to be narrow enough to allow only the fundamental metal–insulator–metal (MIM) plasmonic mode to be coupled. If higher-order MIM plasmonic modes could be coupled to these slits, the problem would become too complex to analyze. Based on these considerations, we fixed the parameters *W*_I_ and *W*_T1_ at 150 nm, which is narrow enough not to allow any other higher-order plasmonic modes but to be reasonably fabricated using the current focused-ion-beam milling process. We also fixed the total thickness of the overall structure (*T*_1_ + *T*_2_) at 300 nm. Therefore, the major remaining parameters for the parameter sweep were the thickness and width of the T-shaped slits (*T*_2_ and *W*_T2_). 

**Figure 4:**
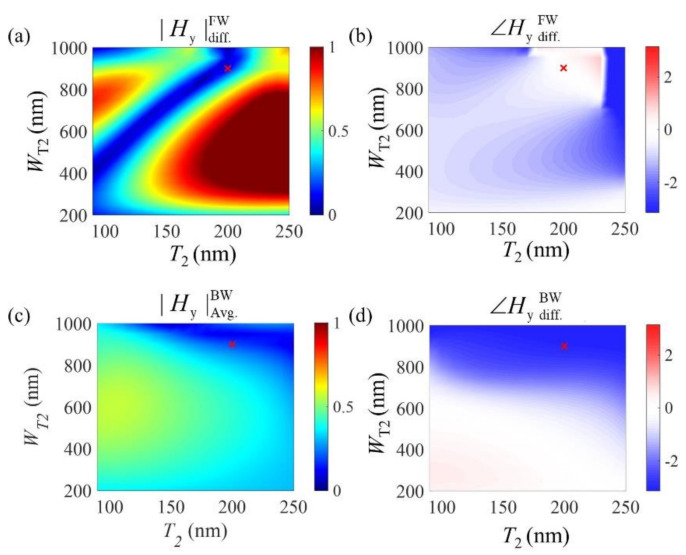
(**a**,**b**) Color maps of *W*_T2_ and *T*_2_ for (**a**) amplitude ($|H_{y}{|}_{\mathrm{diff}.}^{\mathrm{FW}}$) and (**b**) phase differences ($∠H_{y}{}_{\;\mathrm{diff}.}^{\;\mathrm{FW}}$) calculated from the output of the T- and I-shaped slits in the forward illumination case. (**c**,**d**) Calculated (**c**) average of amplitude ($|H_{y}{|}_{\mathrm{Avg}.}^{\mathrm{BW}}$) and (**d**) phase difference between the two slits ($∠H_{y}{}_{\;\mathrm{diff}.}^{\;\mathrm{BW}}$) in the backward illumination case. The red “x” indicates the conditions chosen to optimize the proposed structure.

To best achieve our goal of asymmetric diffraction, the first priority was the suppression of diffraction for the forward illumination case, which could only be achieved when the complex amplitudes from the I- and T-shaped nanoslits were identical for forward illumination. Figure 4a shows the difference in the out-coupled amplitude between the I- and T-shaped slits when the two parameters (*T*_2_ and *W*_T2_) are swept. Similarly, the phase difference characteristics between the two types of slit are shown in Figure 4b. Based on these results, we could determine the appropriate geometric conditions for complex amplitude matching of the out-coupled light from the two types of slits for the forward illumination case, as indicated by the red “x.” The plotted *H*_y_ amplitude and phase values were observed by a detector located 200 nm from the output slits.

Next, the diffraction characteristics of the backward illumination case were analyzed, including those under the optimal conditions determined for the forward direction. The parameter sweeping was performed using the same method except that the illumination was provided from the backward direction, as shown in Figure 4c,d. For the amplitude, we plotted not the difference between but rather the average value of the out-coupled amount from both slits, as the maximum diffraction efficiency would be achieved when the out-coupled lights had a high amplitude and opposite phases. As can be seen from these results, when the light enters in the backward direction, the phase values generated in each slit under nondiffractive conditions (red cross marked in Figure 4d) are not the same as for forward illumination but are shifted almost by a phase of π. Because the parameter sweeping results shown in Figure 4 are only strongly dependent on the variation of *T*_2_ and *W*_T2_ for the forward illumination case, this asymmetric diffraction phenomenon was found to be affected by the surface plasmon polariton excited at the trench of the T-shaped slit. By contrast, because the effects of *T*_2_ and *W*_T2_ are quite small in the backward direction, we were able to separately design each side of the system.

**Figure 5:**
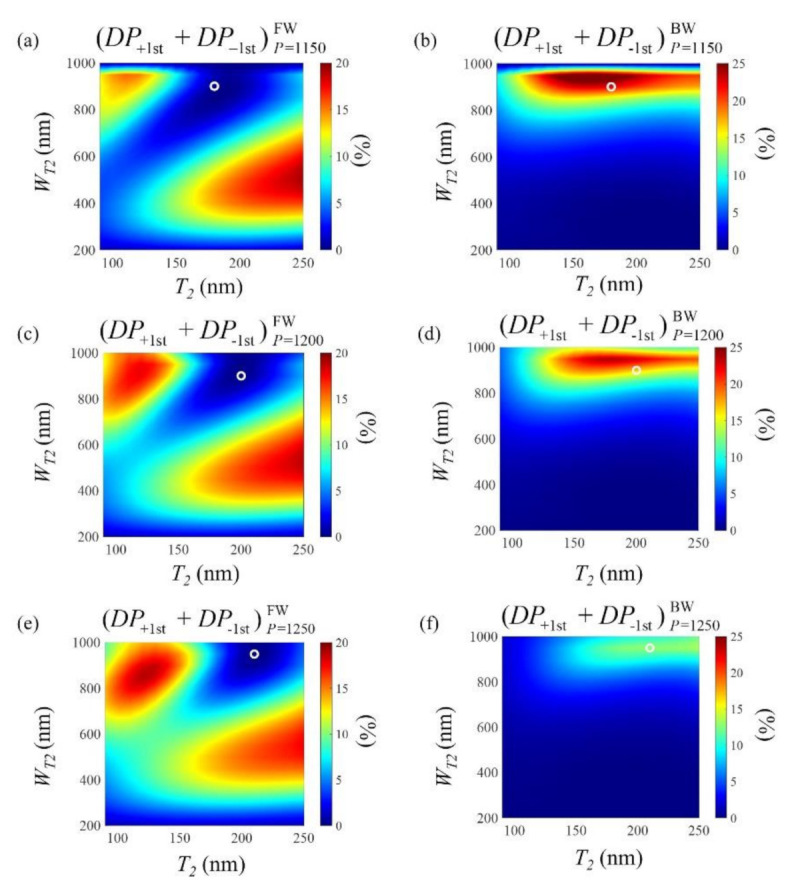
Various conditions for analyzing the sum of plus and minus first-order diffraction power efficiency (*DP*_+1st_ + *DP*_−1st_) according to the metal thickness designed for (**a**,**b**) *P* = 1150 nm, (**c**,**d**) *P* = 1200 nm, and (**e**,**f**) *P* = 1250 nm. (**a**,**c**,**e**) show color images for the forward direction, while (**b**,**d**,**f**) show those for the backward direction. The white circles indicate the optimal thickness at each *P* value of the proposed structure.

In Figure 5, the sum of plus and minus first-order diffraction power efficiency (*DP*_+1st_ + *DP*_−1st_) under several conditions is shown to analyze the diffraction amount of the proposed structure. It is defined as the *z*-directional power coupled to the first-order diffraction versus the input power. We calculated *DP*_±1st_ using Equation (2) by integrating the *z*-component of the complex Poynting vector along the single period:

(2) $$DP_{\pm 1\mathrm{st}}=\frac{\frac{1}{2}\int_{0}^{P}{\left({\vec{E}}_{\pm 1\mathrm{st}}\times {\vec{H}}_{\pm 1\mathrm{st}}{}^{*}\right)}_{z}\;dx}{\frac{1}{2}\int_{0}^{P}{\left({\vec{E}}_{\mathrm{in}}\times {\vec{H}}_{\mathrm{in}}{}^{*}\right)}_{z}\;dx}$$

where ${\vec{E}}_{\pm 1\mathrm{st}}$ and ${\vec{H}}_{\pm 1\mathrm{st}}$ are the complex amplitudes of the 1^st^ order diffracted electromagnetic waves, and ${\vec{E}}_{\mathrm{in}}$ and ${\vec{H}}_{\mathrm{in}}$ are those of incident electromagnetic waves, respectively. Integration of the Poynting vector along a single period, 0 to *P*, will be enough to fully express the total power flow due to the periodic nature of the proposed structure. A similar definition can also be applied to define *DP*_0th_ by replacing 1st order diffracted waves into 0th order diffracted waves.

According to Equation (1), variation of the period can be used to tune the diffraction angle in backward illumination cases. However, as described in Section 2, the $\lambda_{\mathrm{air}}<P<2\lambda_{\mathrm{glass}}$ condition should be satisfied to achieve asymmetric diffraction. Given our free-space wavelength of 980 nm, the period should range from 980 nm to 1306 nm. Three representative periods within this range were selected (1150, 1200, and 1250 nm), and the diffraction amount was determined for each case given variations in *W*_T2_ and *T*_2_.

A similar process for obtaining the optimized conditions was applied to each considered period. First, we determined the complex amplitude matching condition for the forward illumination cases and marked these with white circles. As shown in Figure 5a,c,e, each optimized condition has a near-zero (*DP*_+1st_ + *DP*_−1st_) value, regardless of the period. Meanwhile, Figure 5b,d,f show the (*DP*_+1st_ + *DP*_−1st_) values in the backward direction, which shows reasonably good diffraction performance compared with other nearby geometric conditions.

For the light illumination in the backward direction, several additional analyses were performed within the range of $\lambda_{\mathrm{air}}<P<2\lambda_{\mathrm{glass}}$; the corresponding results are given in Table 1, although these are not outlined in detail. As can be seen from Table 1, we were able to select appropriate design conditions according to the demands of the specific application. When only aiming for a higher transmission {*DP*_0th_ + (*DP*_+1st_ + *DP*_−1st_)} value, the best design condition was *P* = 1100 nm, although in this case, the diffraction angle was still too large. When a reasonable amount of (*DP*_+1st_ + *DP*_−1st_) with a strongly suppressed the *DP*_0th_ was desired, *P* = 1150 nm could be selected. In this way, our proposed structure can be flexibly designed to obtain the desired diffraction angle or diffraction efficiency within the allowable range. As we only studied cases in which some of our geometric parameters, such as *W*_I_, *W*_T1_, and *T*_1_ + *T*_2_, were considered as fixed values, the possibility of finding better conditions with additional degrees of freedom remains.

## 4. Conclusions

In summary, we have proposed a plasmonic metasurface for asymmetric diffraction using an I- and T-shaped nanoslit array. For the proposed metasurface, light with the same amplitude and phase is transmitted from the two types of slits when light illumination is in the forward direction, while the slits are designed to give the opposite phase when light is incident in the backward direction. Therefore, in the case of forward illumination, the optical period *p*_FW_ of the output slits can be considered shorter than the wavelength, whereas that in a backward direction (*p*_BW_) is longer than the wavelength, leading to asymmetrical diffraction based on Huygens’s principle. We expect the proposed structure to be widely applied to diffraction control units for optical waveguide systems, and compact optical asymmetric filters in integrated optics, and it can potentially be used as a part of AR/VR display systems if it is optimized to operate in the visible light range.

## Figures and Tables

**Table 1:** In the case of backward illumination, detailed diffraction results within the periodic condition range satisfying the provision of asymmetric diffraction.

*P* [nm]	*W*_T2_ [nm]	*T*_2_ [nm]	*DP*_0th_ (%)	(*DP*_+1st_ + *DP*_−1st_) (%)	Diffraction Angle (°)
1100	850	190	11.88	15.2	62
1150	900	180	0.80	22.16	58
1200 ^1^	900	200	7.06	16.86	54
1250	950	210	2.42	12.96	51
1300	900	220	0.48	1.84	49

^1^ Periodic condition of the proposed structure.
